# The Unintended Consequences of the Pandemic: The New Normal for College Students in South Korea and Taiwan

**DOI:** 10.3389/fpubh.2021.598302

**Published:** 2021-05-11

**Authors:** Wei-Lin Chen, Sue-Yeon Song, Ko-Hua Yap

**Affiliations:** ^1^Center for Teacher Education, National Sun Yat-sen University, Kaohsiung, Taiwan; ^2^Institute of Distance Education, Korea National Open University, Seoul, South Korea; ^3^Department of Sociology, National Sun Yat-sen University, Kaohsiung, Taiwan

**Keywords:** COVID-19, higher education, college student, lifestyle change, mental health, the new normal, South Korea, Taiwan

## Abstract

This study attempts to compare the impacts of the coronavirus (COVID-19) pandemic on college students' lifestyles and mental health conditions in South Korea and Taiwan. As the COVID-19 outbreak has spread across the globe, it has brought significant changes to college campuses worldwide. College students have been heavily affected by the closure, as online learning has become increasingly common in higher education institutions. Using data collected from college students in South Korea and Taiwan in the spring of 2020, this study examines the effects of pandemic-related lifestyle changes on mental health conditions for college students in the two countries. The results were 3-fold. First, compared to college students in Taiwan, college students in South Korea reported greater decreases in time spent traveling, being with friends, eating at restaurants, and engaging in part-time employment, and greater increases in online shopping and ordering food for delivery. Second, college students in South Korea reported a higher level of worry, a greater possibility of contact with a person with COVID-19, and a lower level of happiness than did college students in Taiwan. Third, our findings indicate that social activities, including spending time with friends, were positively correlated with mental health conditions in South Korea and Taiwan. Comparing Korean and Taiwanese students' lifestyle changes and mental health conditions amid the pandemic, the study argues that the decrease in socialization and interaction under these new circumstances may be a significant factor that explains an increase in mental health issues in Korean college students compared to Taiwanese students, given the increase in confirmed COVID-19 cases in South Korea and the corresponding greater use of online teaching platforms there than in Taiwan.

## Introduction

As the coronavirus (COVID-19) outbreak has spread across the globe, it has had massive social and economic consequences and led to sudden lifestyle changes in the form of social or physical distancing (1). College campuses worldwide have been significantly impacted by the pandemic, as most governments have temporarily closed colleges and universities in an attempt to contain the spread of COVID-19. College students have had to rearrange their daily lives and have been exposed to a completely new campus environment, with a wide variety of modalities being tried across countries. Meanwhile, the pandemic has posed an existential threat to college students' mental health.

An environmental change can result in mental health issues in vulnerable people when environmental stress affects their mood, thinking and behavior. The COVID-19 pandemic, which has disrupted daily life for people worldwide, may put people at greater risk for mental health challenges. Global pandemics cause emotional and health issues, and neuropsychiatric consequences for both infected patients and non-infected individuals (2–5). Studies have identified psychiatric symptoms such as anxiety, stress disorders, and depressive symptoms as consequences of a viral pandemic (5, 6). For example, during the SARS outbreak in Hong Kong, most people felt anxious and changed their social behaviors (6, 7).

Amid the COVID-19 crisis, symptoms of mental health conditions have become a growing concern, and college student populations are not an exception (3). Lockdowns and outbreaks occurring on college campuses may lead to a lack of social support, social and physical isolation, and the disruption of daily routines and activities, increasing college students' mental health problems. According to the results of the Healthy Minds Study survey (3), mental health conditions have affected college students' rates of stress and depression since the start of the pandemic. The report highlighted that over 50% of American college students were concerned about being infected by COVID-19, and nearly 90% were worried about their personal safety and security. Students also expressed a lower level of psychological well-being than they had prior to the outbreak of the virus (3). New research is investigating the effects of COVID-19 on students' mental health, focusing on different country contexts.

In terms of its impact on higher education, COVID-19 has triggered unfavorable mental health outcomes for college students. Studies from different countries have indicated that college students need more support or interventions to cope with stress and uncertainty during the pandemic. Huckins et al. (8) points out that American college students have decreased their physical activity and are going to fewer places while reporting an increase in anxiety and depression symptoms. Focusing on Bangladeshi students, Khan et al. (9) identify stress, anxiety, and depression as common symptoms among college students, with fear of COVID-19 infection as the main causal factor. In addition, studies of Chinese college students indicate that students are worried about their academic delay, negative economic consequences, and routines and activities of daily life (10). However, no studies to date have shown how COVID-19 affects college students' lifestyles and mental health conditions from a comparative perspective, especially in Asia.

This study attempts to compare the impacts of the COVID-19 pandemic on college students' lifestyles and mental health conditions in South Korea and Taiwan. The hypothesis of this study is that students' experiences may vary based on how different institutions and countries have reacted to the pandemic. Therefore, considering the new circumstances created by the COVID-19 crisis and the different policy responses to the pandemic in South Korea and Taiwan, the study aims to understand how lifestyle changes have impacted students' mental health in Korea and Taiwan during the COVID-19 pandemic.

Using data collected from college students in both countries, this study explores differences and similarities across the following three aspects of their experience in relation to the threat of COVID-19. First, we investigate whether students in the two countries face different issues related to mental health. Second, we examine whether students in the two countries have different patterns of lifestyle changes. Third, we examine whether the extent of lifestyle changes contributes to the mental health of college students in the two countries. In doing so, the study tries to examine the different scenes on college campuses and compare the unintended consequences of the pandemic for college students' lives in the selected countries from a comparative perspective.

## Policy Responses to COVID-19 in Korea and Taiwan

Until last August, both South Korea and Taiwan were among the few countries that had demonstrated success in curtailing the spread of the virus by adopting necessary measures to mitigate the impact of subsequent outbreaks (11). Capitalizing on their prior experience with SARS in 2002–2003 and MERS in 2015, both countries exhibited robust and consistent standard operating procedures (12). The governments' decisive actions early in the crisis achieved favorable outcomes, flattening the curve in both countries (13). More recently, the early gains in South Korea have given way to alarm as the country has faced an unstoppable wave of infections (14).

Taiwan took aggressive action to combat the outbreak as soon as the WHO reported the existence of a virus of unknown cause in Wuhan, China. Taiwan immediately closed off all travel from China, activated its Central Epidemic Command Center, began screening arrivals, and deployed detailed contact tracing, even before the World Health Organization advised such a step (15). The Entry Quarantine System was also launched, requiring travelers to complete a health declaration detailing their travel histories, specific symptoms, and health evaluations (16). Travelers were mandated to complete a 14-day home quarantine, which included self-isolation without going out or having visitors and recording temperature twice a day (17). Travel restrictions were implemented, and only those foreigners holding Alien Resident Certificates were allowed into the country (18). Finally, the government disseminated a health promotion message recommending handwashing routines and mask-wearing in crowded or enclosed places (17).

In South Korea, the situation was more challenging, as the country reported the second-highest number of confirmed cases of the virus after China between January and February when a cluster of cases was identified in Daegu, a city of ~2.5 million (11). After this peak, the number of confirmed cases declined rapidly, although occasional minor resurgences continued to occur. Korea's response to COVID-19 was seen as successful, as it was one of the first countries to quickly flatten the curve. Korea managed to mitigate the pandemic by implementing widespread testing, contact tracing, and quarantines for all positive patients (15). Beginning in the early stages of the major outbreak, the government collaborated with the scientific community and directed companies to produce a diagnostic reagent. In April, Korea expanded its testing capacity to provide an average of 15,000 diagnostic tests per day (19). However, the virus spread through local communities, increasing the chances that the virus would spread over a wider part of the country, and the government announced that Level 2 social distancing, the second highest tier in a three-tier system, would be implemented in the capital area beginning in August 2020 (20).

The Korean government also adopted a nationwide contact-tracing program that combined traditional shoe-leather epidemiology with new methods that make efficient use of technology and large databases (i.e., global positioning systems, credit card transactions, and closed-circuit television) (21). People identified as having had contact with confirmed or suspected cases were asked to self-quarantine at home or in designated facilities, and as in Taiwan, mandatory 14-day quarantines were required for all travelers entering the country (19).

## Higher Education Responses to COVID-19 in Korea and Taiwan

In response to the COVID-19 outbreak, Korea and Taiwan moved quickly to order their populations to stay at home, practice handwashing, engage in social distancing, and wear masks in public settings (22, 23). Given this situation resulting from the global pandemic, college students have experienced a “new normal” in the higher education environment. The International Association of Universities (IAU) survey report on the impact of COVID-19 on higher education institution (HEI) highlighted that one of the key challenges encountered by HEIs was the sudden shift to distance learning (24). The results of the report revealed that over 50% of HEIs across the globe made transitions from classroom teaching to distance learning. The rate of change varied by region, e.g., 85% in Europe, 72% in the Americas, and 60% in Asia. HEIs in different regions explored various formats for learning; some colleges and universities continued face-to-face learning, some explored blended or hybrid learning, and some went primarily online with some in-person courses or went fully online with no students on campus (25).

Colleges and universities in Korea and Taiwan took proactive actions, implementing different levels of restrictions to secure the safety of students. For example, college students in South Korea were temporarily restricted from campus facilities, and all courses switched to an online format in the spring of 2020. As remote learning became prevalent on campuses, the frequency of face-to-face interactions with peers and faculty decreased tremendously for Korean college students. Taiwan, on the other hand, was one of the few countries in which campuses remained in session, due to the virus appearing to be under control. In Taiwan, the government established guidelines to secure the safety of students and staff, while colleges and universities remained open during the spring of 2020. Taiwanese students were required to wear a facemask, maintain social distancing in the classroom, and check their body temperature on a daily basis. The guidelines, including measures of self-management of health, quarantine, and regulations on school assemblies, also reduced opportunities for interacting with peers and faculty among Taiwanese college students (22).

As campus lockdown restricted opportunities for socialization and interaction among students and faculty on campus, college students' mental health became a special challenge during COVID-19 (26). Previous studies have pointed out that the campus environment is where socialization occurs, and also where students gain knowledge, integrate skills, and develop the capacity to cope with challenges in society (27). The new normal of non–face-to-face learning on campuses has limited college students' opportunities for physical interaction with peers and faculty. Thus, the pandemic had the unintended consequences of decreasing college students' opportunities to develop their capacities and resilience (28), causing an existential threat to their mental health. To better understand the ways that COVID-19 has impacted college students' lifestyles, the following research questions were asked:

Research Question 1: Have college students in South Korea and Taiwan presented different patterns of mental health during the COVID-19 pandemic?

Research Question 2: Have college students in South Korea and Taiwan presented different patterns of lifestyle change during the COVID-19 pandemic?

Research Question 3: How have students' lifestyle changes determined their mental health during the pandemic in South Korea and Taiwan?

## Participants and Methods

### Participants

Data were collected from college students in both South Korea and Taiwan between May and June of 2020. Participants were selected from two institutions, one in Seoul, South Korea and the other in Kaohsiung, Taiwan, each of which is a preeminent research university located in a big city. All college students in the Korean case were surveyed with convenience sampling using an e-mail invitation to an online survey that was sent to all students through the university's online system due to the campus lockdown and the enforced use of online learning platforms. In the Taiwanese case, participants were limited to college seniors and recruited using probability sampling and in-person interviews. International students were eliminated from the analysis. A total sample of 554 South Korean college students and 335 Taiwanese college students completed the survey. There were some similarities between the two institutions. For example, over 50 percent of participants were male in both South Korea (50.4%) and Taiwan (58.8%). In addition, most students were enrolled in STEM majors, including 53% of Korean students and 59% of Taiwanese students. There were also differences among the participants in the two selected countries; most of the Korean students' parents had received a bachelor's degree or higher (86%), while less than half (44%) of the Taiwanese students' parents had done so. More than half (52%) of the Taiwanese respondents lived off campus, with another 40% in on-campus dorms and 8% at home. In the Korean sample, 56% reported living at home, 30% off campus, and 14% in an on-campus dorm.

### Measures

Three aspects of mental health were assessed in the study, including “worry,” “risk of contact,” and “happiness.” These three measures reflect key aspects of mental health, and have often been used in studies to determine mental health conditions during a pandemic (29–31). To measure the first indicator “worry,” we asked students “*On a scale from 0 to 10, please rate how worried you are about COVID-19*.” The second variable, “risk of contact” was assessed using the question, “*On a scale from 0 to 10, please rate the possibility of contacting with a person known to have COVID-19*.” The third question measured happiness using a 4-point Likert scale that asked, “*How would you say things are these days—would you say that you are not at all happy, not too happy, fairly happy, or very happy?*” All three variables were treated as continuous in the analysis.

Seven measures of lifestyle change adapted from the existing literature were assessed to understand how COVID-19 changed students' lifestyles (30, 32). Students were asked, “*Compared to before the COVID-19 outbreak, how has your lifestyle changed?*” with regard to seven aspects of lifestyle, including traveling, spending time with friends, eating at restaurants, getting restaurant takeout, getting food delivered, having part-time jobs, and shopping online. The response options used a 5-point scale to allow the individual to express the change in frequency of each event. The scale responses were “decreased a lot,” “decreased a little,” “no change,” “increased a little,” or “increased a lot.” All measures were treated as continuous variables indicating the frequency of lifestyle changes.

The questionnaire also collected data on sociodemographic characteristics, including gender, college major, parental education level, and living arrangements.

### Statistical Analysis

To analyze the effects of lifestyle changes on students' mental health during COVID-19, we conducted the following three analyses. First, descriptive statistics of the key variables of the analytic sample were provided. Second, a *t*-test was used to examine whether the main items were significantly different between South Korea and Taiwan. Third, regression analyses were performed to examine the association between lifestyle changes and three aspects of mental health. All statistical regressions controlled for gender, parental educational level, college major, and living arrangement.

For the Taiwanese data, <1% of cases were missing, and listwise deletion was applied in the analysis. The Korean sample included no missing values. All analyses were conducted using Stata/MP16.1.

## Results

### Mental Health During COVID-19

Table 1 presents descriptive statistics for all variables used in the study. The results showed that South Korean college students were more worried about COVID-19 (*M* = 6.487; SD = 2.518) than Taiwanese college students were (*M* = 4.02; SD = 2.25). Additionally, the mean response for the possibility of having contact with someone with COVID-19 was 5.60 ± 2.36 in South Korea, but it was 3.05 ± 2.02 in Taiwan. Finally, the mean happiness score was 2.67 ± 0.78, compared to 2.89 ± 0.60 in Taiwan. The results from the *t*-test also indicated that South Korean college students presented a relatively higher level of mental health concerns than Taiwanese college students did.

**Table 1:** Descriptive statistics.

**Variable**	**South Korea**		**Taiwan**		
	**Mean**	**SD**	**Mean**	**SD**	***t***
**Dependent variables**					
Worry	6.49	2.52	4.01	2.25	−15.17^***^
Risk of contact	5.60	2.36	3.05	2.02	−17.08^***^
Happiness	2.67	0.78	2.89	0.60	4.75^***^
**Independent variables**					
Life Change					
Travel	1.23	0.59	2.07	0.79	16.82^***^
Hangout with friends	1.69	0.71	2.79	0.54	25.90^***^
Eating at a restaurant	2.01	0.86	2.41	0.76	7.27^***^
Restaurant takeout	3.35	1.08	3.54	0.76	3.13^**^
Food delivery	3.85	1.06	3.41	0.72	−7.31^***^
Part-time jobs	2.13	1.05	2.99	0.54	16.01^***^
Online shopping	3.68	1.00	3.16	0.50	−10.42^***^
Major	%		%		
Liberal Arts	18.4%		17.6%		
STEM	52.5%		59.4%		
Business	4.5%		15.8%		
Social Sciences	24.6%		7.2%		
Gender					
Male	50.4%		58.8%		
Female	49.6%		41.2%		
Parental Education Level					
HS or less	7.9%		25.7%		
Some college	6.3%		30.2%		
BA	49.3%		20.3%		
Advanced	36.5%		23.9%		
Living Arrangement					
Home	56.0%		7.8%		
Dorm	14.3%		40.3%		
Outside the campus	29.8%		51.9%		
*N*	554		335		

* *p < 0.05*,

** *p < 0.01*,

*** *p < 0.001*.

**Figure 1:**
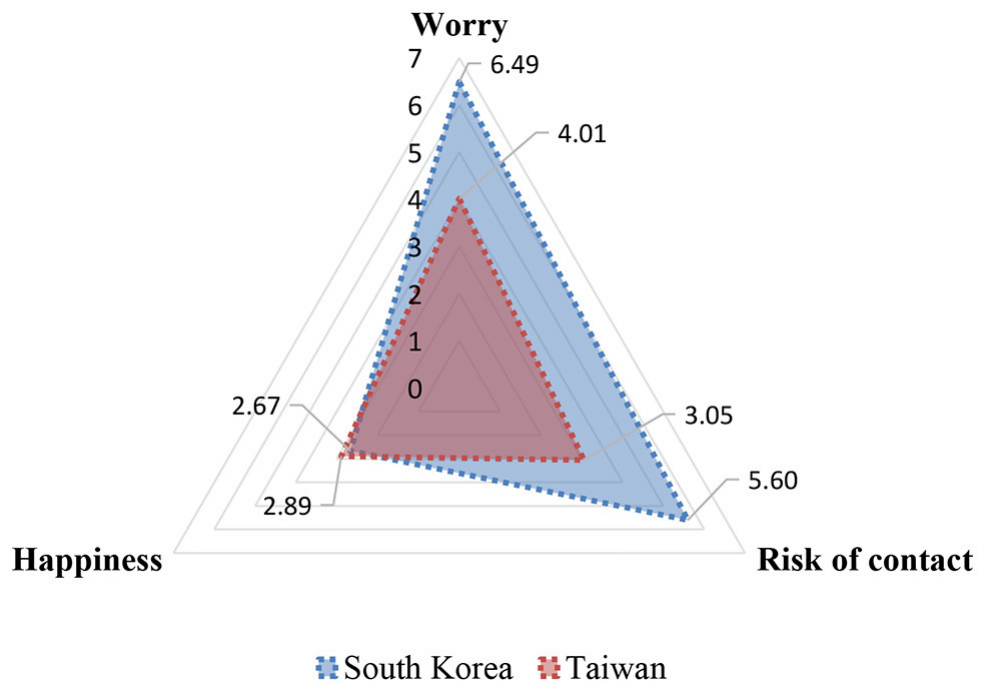
Comparison of mean scores for three aspects of mental health conditions.

To visualize differences in mean scores between the two selected countries with regard to the main outcome variables used in the study, Figure 1 presents information for each item.

### Lifestyle Changes During COVID-19

To investigate the different patterns of lifestyle changes, the survey questions inquired about college students' daily experiences during the COVID-19 lockdown. The two countries presented variations in their lifestyle changes resulting from the COVID-19 pandemic (see Table 1). Compared to Taiwanese students, South Korean college students significantly decreased their frequency of traveling (M = 1.23; SD = 0.59), spending time with friends (M = 1.69; SD = 0.71), eating at restaurants (M = 2.01; SD = 0.86), and working part-time (M = 2.13; SD = 1.05). Additionally, the results from the *t*-test indicated an increased frequency of food delivery (M = 3.85; SD = 1.06) and online shopping (M = 3.68; SD = 1.00) in South Korea compared to Taiwan (frequency of food delivery: M = 3.41; SD = 0.72; frequency of online shopping: M = 3.16; SD = 0.50).

### Effects of Lifestyle Changes on Mental Health

To examine the relationship between lifestyle changes and three aspects of mental health, multiple regression analyses were conducted while controlling for gender, parental education level, living arrangement, and college major. For South Korea, age was also included as a control variable, since the data sample included undergraduates between 19 and 29. The age distribution of undergraduates ranged between 19 and 29 in the sample because in South Korea, the majority of college-aged male citizens are required to serve at least 21 months in the military, choosing whether they will suspend their undergraduate work during their years in college or take off immediately after graduation to serve in the army.

To assess the robustness of the findings, this study conducted a series of sensitivity analyses for Korean sample using senior college students only and also performed sets of analyses without controlling for age in each model. The overall results were similar to those based on primary analyses. The results of each model are reported in Table 2.

**Table 2:** Results of multiple regression analyses on three aspects of mental health conditions.

**Variable**	**Worry**		**Risk of contact**		**Happiness**	
	**Korea**	**Taiwan**	**Korea**	**Taiwan**	**Korea**	**Taiwan**
**Life change**							
Travel	−0.215 (0.185)	−0.299 (0.170)	−0.039 (0.182)	−0.076 (0.157)	−0.096 (0.061)	0.037 (0.048)
Hangout with friends	−0.625^***^ (0.169)	−0.198 (0.234)	−0.419^*^ (0.167)	0.282 (0.216)	0.138^*^ (0.056)	0.017 (0.066)
Eating at a restaurant	−0.231 (0.131)	0.002 (0.189)	−0.182 (0.129)	0.243 (0.174)	−0.010 (0.043)	−0.076 (0.054)
Restaurant takeout	−0.000 (0.101)	0.500^**^ (0.190)	0.019 (0.100)	0.161 (0.174)	0.102^***^ (0.033)	0.004 (0.054)
Food delivery	0.070 (0.105)	−0.105 (0.183)	0.059 (0.103)	−0.157 (0.168)	−0.074^*^ (0.035)	−0.007 (0.052)
Part-time jobs	−0.391^***^ (0.103)	−0.807^***^ (0.224)	−0.287^**^ (0.102)	−0.169 (0.206)	0.017 (0.034)	0.150^*^ (0.063)
Online shopping	0.298^**^ (0.105)	0.335 (0.244)	0.124 (0.103)	0.716^**^ (0.224)	0.034 (0.035)	0.077 (0.069)

*Standard errors in parentheses*.

* *p < 0.05*,

** *p < 0.01*,

*** *p < 0.001*.

*All models include gender, age (Korea only), parental education level, living arrangement, and college major as control variables*.

First, standard multiple regression analyses were performed to investigate the effects of lifestyle changes on worry during COVID-19 (see Table 2). The results show that among South Korean college students, an increasing frequency of spending time with friends and working part-time was negatively correlated with being worried about COVID-19. In other words, when students had a higher level of worrying about COVID-19, they were more likely to decrease the time they spent with friends and engaged in part-time employment. Additionally, an increased frequency of online shopping was positively correlated with being worried about COVID-19. Students who were more worried about COVID-19 were more likely to go shopping online. Among Taiwanese college students, the pattern of part-time jobs was similar to that of South Korean college students, reflecting the negative relationship between part-time employment and being worried about COVID-19. The results of Taiwanese college students also presented a positive correlation between getting restaurant takeout and worrying about COVID-19. Students who were more worried about COVID-19 increased their frequency of getting restaurant takeout.

Second, we investigated the relationship between college students' lifestyle changes and their risk of contact with someone who has COVID-19, as demonstrated in Table 2. In Korea, the increasing frequency of spending time with friends and working part-time was negatively correlated with the self-reported risk of having contact with someone who has COVID-19. College students in Korea considered spending time with friends and working part-time to decrease the possibility of contracting COVID-19. However, Taiwanese college students presented different patterns. In Taiwan, the increasing frequency of online shopping was positively correlated with a self-reported risk of contact with someone who has COVID-19. Since the result indicated a relation and not causality, reverse causality existed between online shopping and the risk of contact with someone who has COVID-19. Taiwanese college students who reported a higher risk of contact with someone who has COVID-19 may go shopping online more often.

Third, the results predicting life changes and happiness indicated different patterns in both South Korea and Taiwan (see Table 2). In South Korea, the increasing frequency of spending time with friends and getting restaurant takeout was positively correlated with happiness, but food delivery was negatively correlated with happiness. During the COVID-19 pandemic, spending time with friends increased happiness among South Korean college students, reflecting the important role of social support from peers. The positive relationship between restaurant takeout and happiness also indicated the importance of having contact with other people during the COVID-19 pandemic in South Korea. In Taiwan, the increasing frequency of working part-time was positively correlated with happiness, reflecting that part-time employment increased levels of happiness as well as reflected the importance of interacting with other people during the COVID-19 pandemic.

Finally, statistically non-significant relationships between college students' lifestyles and mental health conditions were also presented in Table 2. The non-significant findings indicated the various role of life changes in predicting different aspects of mental health conditions in both South Korea and Taiwan. For example, both traveling and eating at restaurants were statistically non-significant with mental health conditions (i.e., worry, risk of contact, and happiness). Getting restaurant takeout was statistically non-significant with the possibility of contracting COVID-19. The frequency of food delivery was statistically non-significant with both worry and risk of contact. The frequency of online shopping may not increase college students' happiness, since the relationship between online shopping and happiness was statistically non-significant.

## Discussion and Conclusion

The purpose of the study was to examine the correlation between lifestyle changes and mental health among college students in South Korea and Taiwan during the COVID-19 pandemic. HEIs have been significantly disrupted, with millions of students around the world studying remotely due to campus closures (33). There is no clarity as to how COVID-19 will impact the overall operations of HEIs in upcoming semesters; however, what we clearly know is that this pandemic has produced some unexpected changes in the higher education community. In this regard, it is necessary to understand emerging patterns of lifestyle changes caused by the pandemic and college students' responses to their new experiences and mental health consequences of COVID-19. The main findings of the study are as follows.

First, we explored whether lifestyles changed among college students confronting a “new normal” in the two selected countries given the serious global health threat caused by the COVID-19 pandemic. College students in South Korea indicated a decrease in traveling, spending time with friends, eating at restaurants, and part-time employment, and an increase in food delivery and online shopping compared to college students in Taiwan. During the pandemic, Korean students significantly decreased their daily activities, as did many in other countries, while students in Taiwan experienced less lifestyle change (8). Second, we examined different patterns of mental health among students in the two countries. Similar to findings from Bangladesh, China, and the U.S. (3, 8–10), college students in South Korea reported a higher level of worry, a higher possibility of having had contact with someone with COVID-19, and a lower level of happiness than before the pandemic. However, Taiwanese college students presented a different pattern, with a higher level of happiness than the South Korean students. A possible explanation could be that students in South Korea were temporarily restricted from campus facilities, while campuses remained open in Taiwan as the pandemic was under greater control there (22). Since campus lockdown restricted the opportunities for socialization and interaction on campus in South Korea (26), college students there may have struggled with mental health problems, feeling unsafe and anxious during COVID-19 (5, 6). The results from South Korea indicate that environmental changes such as those that occurred during the global pandemic can cause emotional and health issues even among non-infected individuals (2–5). Third, we discovered how different levels of lifestyle change have contributed to the mental health of college students in the two countries. The results indicated that social activities, including spending time with friends, were positively correlated with mental health in South Korea and Taiwan. The positive correlation between mental health and social activities in both South Korea and Taiwan confirmed the important role of the campus environment in developing students' capacity to cope with challenges in society (27). College students in South Korea who increased the frequency of time spent with friends were less worried about COVID-19, reported a lower possibility of having had contact with a person known to have COVID-19, and indicated a higher level of happiness. College students in Taiwan were less worried about COVID-19 if they increased the frequency of part-time employment.

In this study, we tried to investigate the unintended consequences of COVID-19 on college students' lives, assuming that their experiences might vary based on how different institutions and countries have reacted to the pandemic. There were different patterns of policy and institutional responses to COVID-19 among HEI in South Korea and Taiwan (22–24). College students in Korea were required to stay at home, take online courses, or maintain social distancing during the pandemic, while students in Taiwan remained onsite with safety measures implemented on campus. Given the increase in confirmed COVID-19 cases and use of online teaching platforms in South Korea (11, 15), this may be the main factor explaining why Korean college students experienced more disruption of their daily routines and more mental health issues than Taiwanese college students. Finally, since both countries implemented a series of policy/program actions in response to the virus (15–17), college students in both South Korea and Taiwan decreased the frequency of daily activities and had fewer opportunities for socialization and interaction with peers and faculty under the new circumstances created by the COVID-19 crisis.

As the number of confirmed cases has continued to increase, governments and HEIs have taken more aggressive actions against the COVID-19 pandemic, including year-long campus lockdowns, temporary closures, and virtual learning formats (22). Campus lockdowns and online learning formats are aimed at reducing the possibility of physical contact during the pandemic; however, college students are receiving less emotional and social support from peers and colleagues who are self-isolating at the same time. This study highlights the positive relationship between social support and interaction with friends and mental health conditions. In line with previous studies that specified the importance of perceived social support in reducing mental health problems (34–36), our findings shed light on the importance of social and institutional support for college students in reducing the incidence and prevalence of some mental disorders during the pandemic, and suggest that more interventions and support from policy/program perspectives are needed.

The comparison between South Korea and Taiwan with respect to college students' mental health can benefit administration officials and policymakers as they implement policies and practices addressing the aftermath of the pandemic. Governments and HEIs across countries need more empirical evidence to balance safety and learning for college students. We suggest that governments and HEIs organize social support activities through online or hybrid formats, since students' mental health is more vulnerable during COVID-19. Various social activities and forms of social support benefit the learning development and mental health among college students.

Along with these contributions, some limitations exist in our study. Considering the time constraints and data availability, the study was limited to two institutions from each of the selected countries. The results should be cautious while generalizing to the entire population in the selected countries. More research is needed in this unprecedented time to share insightful implications from various country contexts; empirical studies with nationally representative longitudinal datasets are also needed to support college students in maintaining their academic path in a safe manner.

## Data Availability Statement

The raw data supporting the conclusions of this article will be made available by the authors, without undue reservation.

## Ethics Statement

Ethical review and approval was not required for the study on human participants in accordance with the local legislation and institutional requirements. Written informed consent for participation was not required for this study in accordance with the national legislation and the institutional requirements.

## Author Contributions

WLC and SYS: conception and design, analysis and interpretation of the data, drafting of the article, critical revision of the article, and administrative, technical, or logistic support. WLC, SYS, and KHY: final approval of the article and provision of study materials. All authors contributed to the article and approved the submitted version.

## Conflict of Interest

The authors declare that the research was conducted in the absence of any commercial or financial relationships that could be construed as a potential conflict of interest.
